# Educators’ Perspectives of Working Conditions in Inclusive Elementary Schools

**DOI:** 10.1177/00222194251325857

**Published:** 2025-03-17

**Authors:** David DeMatthews, Elizabeth Bettini, Bonnie Billingsley, Emily M. Burns

**Affiliations:** 1 University of Texas-Austin, USA; 2 Boston University, MA, USA; 3 Virginia Tech, Blacksburg, USA; 4 Kent State University, OH, USA

**Keywords:** elementary school, inclusion, working conditions, teacher, principals, students with disabilities

## Abstract

Educators need supportive working conditions to fulfill their responsibilities to students, families, and colleagues. Given the crucial role of working conditions in teacher effectiveness, we sought to understand educators’ (including general educators, paraeducators, specaial educators, and principals) perspectives about their working conditions as they included students with disabilities. We analyzed 28 primarily qualitative studies, conducted between 1998 and 2023, using Conservation of Resources (COR) theory as a framework to study their working conditions. We analyzed their responsibilities in inclusive schools (e.g., instruction, collaboration), and the resources that were provided or needed to fulfill those responsibilities (e.g., time, professional development). We found inclusion often required substantial responsibilities for educators; however, they often lacked needed resources, leaving them feeling stretched thin as they tried to meet students’ needs. These findings have implications for supporting educators in inclusive schools.

Students with disabilities, including those with learning disabilities (LDs), are increasingly served in general education classrooms (Williamson et al., 2020), substantially changing educators’ responsibilities (Bettini et al., 2022). Although special education teachers (SETs) used to have primary responsibility for serving students with disabilities in separate spaces, SETs, general education teachers (GETs), principals, paraeducators, and related service providers now share responsibility for students with disabilities (DeMatthews et al., 2023). To fulfill these responsibilities, educators (including principals, paraprofessionals, and other school personnel) depend on working conditions that foster their capacity to effectively include students with disabilities. Working conditions include both the responsibilities educators are assigned (e.g., instruction, planning) and the resources available to fulfill those responsibilities (e.g., social support, time). Although research on working conditions is growing (e.g., Stark et al., 2023), less is known about educators’ conditions in inclusive schools.

A growing body of research indicates working conditions contribute to the quality and effectiveness of the services educators provide. For example, Ronfeldt et al. (2015) found teachers were more effective at promoting student achievement gains when working in schools with more collaborative cultures. Using mixed methods, Mathews et al. (2021) found SETs provided higher quality reading instruction in self-contained settings when they collaborated with skilled colleagues and trained paraeducators. However, these studies focused on GETs (e.g., Kraft & Papay, 2014) or SETs in separate settings (e.g., Siuty et al., 2018). To our knowledge, no studies examined how working conditions shape educators’ efforts to serve students with disabilities in inclusive schools (Stark et al., 2023).

To address this need, we reviewed research on inclusive elementary schools from 1998 to 2023 to understand the educators’ working conditions as they served students with disabilities in inclusive settings, including students with LD. Although these studies did not focus primarily on working conditions, they provide insights into conditions that, from educators’ perspectives, facilitated or constrained their efforts to serve students with disabilities. Understanding how educators’ working conditions shaped their efforts in inclusive schools has implications for improving support, and thus improving students’ experiences and outcomes. We first provide background on inclusive schools and second, describe Conservation of Resources (COR) theory (Hobfoll et al., 2018), which we used to define working conditions and structure our analyses.

## Background and Context: Inclusive Schools

Educators who work to create and improve inclusive schools do so in messy contexts, shaped by complex local and national histories, policies, and practices of both exclusion and advocacy for inclusion. Students with disabilities were historically excluded from most public schools, partly rooted in ableist assumptions that students with disabilities were uneducable and unworthy of investment (Osgood, 2009). The landmark *Brown v. Board of Education* (1954) Supreme Court decision provided a legal foundation for disability rights groups to secure key federal court victories, culminating in the passage of the Education for All Handicapped Children Act (EAHCA, 1975). EAHCA first established that public schools could not deny an education to any student with a disability, regardless of cost or the nature of their disability.

Yet, EAHCA (1975) and its reauthorizations (the Individuals with Disabilities Education Act [IDEA]) have never included the words “inclusion” or “inclusive”; rather, Congress endorsed the “least restrictive environment” (LRE) principle, which establishes a presumption of placement in general education settings but also a continuum of other placements (e.g., resource, self-contained). In interpreting LRE, no federal court has ever held that inclusion in general education settings was required or a right (Zirkel, 2020).

Nevertheless, the inclusion movement has pushed schools to become more inclusive (e.g., Stainback & Stainback, 1984), sometimes using the proportion of students in general education for more than 80% of the day as a benchmark for assessing the success of these reforms (e.g., McLeskey et al., 2014). By this metric, schools have become substantially more inclusive, as 66% of students with disabilities now spend more than 80% of their day in general education settings, including 75% of students with LD (U.S. Department of Education, 2024). Yet, this growth has not been equitably distributed to all students; for example, Black and American Indian students remain at higher risk of being placed in segregated settings and suspended or expelled relative to their White peers (Grindal et al., 2019; U.S. Department of Education [U.S. DOE], 2023).

Although the 80% metric is often used as an indicator of inclusion, many researchers have pushed back on the notion that placement in a general education class, by itself, constitutes inclusion. McLeskey et al. (2022) defined inclusive schools as “places where students with disabilities are valued and active participants and where they are provided supports needed to succeed in the academic, social, and extra-curricular activities of the school” (p. 4). The definition highlights the importance of inclusive schools attending to students’ strengths and support needs. Adopting a more critical definition that recognizes ableism, racism, classism, and other historically rooted systems of oppression as core to exclusionary policies and practices, Waitoller and Artiles (2013) defined inclusion as a “systemic process of overcoming barriers to participation and learning for all students,” (p. 327), including both students with disabilities and other marginalized students.

Educators working to implement inclusion may adopt their own definitions of the term, shaped by broader discourses and by their own state, school, and district contexts (Rogers, 2002). Thus, understanding inclusion requires understanding the contexts in which it is enacted—including local, state, and national policies and practices. For example, state education agencies and legislatures are responsible for funding public education, creating a system of continuous improvement, and implementing IDEA, including LRE; yet, only 22 states received the U.S. DOE’s (2023) “Meets Requirements” designation for implementing IDEA. Likewise, some states do not adequately or equitably fund public schools (Baker et al., 2021), constraining educators’ opportunities to engage in inclusive reforms or sustain high-quality teachers. The working conditions in which educators enact inclusion are shaped by these broader contexts.

## Conceptual Framework and Research Purposes

We used COR theory to conceptualize working conditions (Hobfoll et al., 2018). COR theory posits that employees meet job demands (i.e., responsibilities) by deploying their resources, experiencing stress when demands exceed resources to fulfill responsibilities well (Hobfoll et al., 2018). COR theory has been used to study employee outcomes in many professions, with meta-analyses showing how the balance of responsibilities and resources predicts stress, burnout, and other psychological outcomes (Alarcon, 2011; Hobfoll et al., 2018).

Growing research has used COR theory to conceptualize SETs’ working conditions (e.g., Bettini et al., 2020). Several studies focused on educators’ responsibilities and the resources they use to fulfill those responsibilities, including social (e.g., colleague support), logistical (e.g., time), and informational (e.g., coaching) resources (e.g., Bettini et al., 2020; Billingsley & Bettini, 2017; Mathews et al., 2021). These studies found that the balance between SETs’ responsibilities and resources contributes to their instructional quality and retention. Informed by COR theory, the purpose of this synthesis is to understand the working conditions of educators (i.e., principals, SETs, GETs, paraeducators, related services personnel) as they served students with disabilities in inclusive settings. Specifically, we examine educators’ perspectives on their responsibilities in inclusive settings, and resources on which they relied. Understanding these conditions has implications for how leaders, educator preparation programs, and policymakers support educators in inclusive schools.

## Method

### Inclusion and Exclusion Criteria

We included studies published between 1998 and August 29, 2023. We chose to begin after the 1997 reauthorization of IDEA, which emphasized increased access to the general education curriculum for all students with disabilities, thus reinforcing the general movement toward greater inclusion of students with disabilities in general education classes (Williamson et al., 2020). We included only studies in the United States—given varied national policies—and studies focused on public inclusive schools or classes, or those working to be inclusive. We did not impose any criteria for what constituted inclusion but, rather, deferred to authors’ claims about these settings (An & Meaney, 2015). Criteria for inclusion also ranged across the studies. One study selected participants only if they focused on students identified as having a developmental disability and a history of engaging in challenging behaviors that might be considered disruptive to the learning of others (Lohrmann & Bambara, 2006). Another study only selected principals if their schools showed progress in providing students with disabilities increased access to the general education classroom during their leadership tenure (DeMatthews & Mawhinney, 2014).

We excluded studies not focused on public inclusive schools or classes, even if students with disabilities were served in general education classes. We only included studies in elementary schools, as elementary and secondary schools are organized differently, with differences in their working conditions; we included studies across multiple school levels, if results were disaggregated for elementary (e.g., Kent-Walsh & Light, 2003). We excluded studies of educators’ attitudes toward inclusion and preservice preparation. As inclusive schools depend on coordination of effort among many educators (SETs, GETs, principals, related service providers; Bettini et al., 2022), we included studies regardless of which educators participated. We only included studies that provided insights into educators’ perspectives on working conditions, substantiated by quotes, observations, or quantitative data. We included studies regardless of the disability categories under which students qualified for services.

We included studies using any methodology (qualitative, mixed methods, quantitative). We did not screen out less rigorous studies, though we critiqued methodological strengths and weaknesses. We excluded studies where participant population, data collection methods, or analysis methods were not stated.

### Search Procedures

We searched using the Education Research Complete database from 1998 to August 29, 2023, in consultation with a research librarian. We searched peer-reviewed articles’ keywords, titles, and abstracts using a Boolean phrase, with terms reflecting (a) educators’ roles (e.g., principal, teacher), (b) disability (e.g., disab*), (c) inclusion (e.g., inclus*), and (d) elementary (see Appendix). The initial search yielded 998 studies, after removing duplicates. To build consensus about inclusion/exclusion criteria, the first three authors coded the same 50 studies. We then coded another 50 studies, with 92% agreement related to the inclusion/exclusion criteria described above. We divided the remaining 898 studies, with each author reviewing their group of studies for inclusion/exclusion. We discussed questions until we reached consensus, which yielded 17 studies. We found 9 more studies from an ancestral search of the 17 initial articles and 2 more from a literature review, resulting in 28 studies.

### Analysis of Studies

We analyzed the results of this review in four steps. First, the first three authors read each study, completing detailed summaries describing research questions/purposes, inclusion definition, theory used, methods, results, key quotes, and a study critique. Second, we developed a matrix, based on COR theory, of responsibilities and resources; the fourth author deductively coded findings from article summaries in the COR matrix form. For responsibilities, we coded for instructional, administrative, collaboration/support, and other responsibilities. For resources, we coded for social, logistical, informational, and other resources. In addition, we also described any relevant descriptions of how working conditions affected outcomes (e.g., insufficient resources and limited collaboration left some teachers feeling unsupported [Ko & Boswell, 2013]). At least two authors reviewed each study and reviewed the coded findings. Third, we used both the summaries and completed matrices to identify themes and subthemes, returning to the original studies as needed. Fourth, the first three authors drafted specific sections, reading and reviewing all sections multiple times. We also met regularly throughout the 8-month process to discuss our progress, findings, and discussion, with all members present in most meetings. During these regular meetings, the research team shared interpretations and experiences, and engaged in critical questioning until the manuscript was finalized.

## Findings

### Overview of Studies

We found 28 studies over a 26-year period that described the perspectives of elementary principals and educators as they worked in inclusive schools, providing insights into their working conditions. Studies ranged widely in their foci, including studies of inclusive school reform in one or more schools (e.g., Theoharis et al., 2016), the sustainability of inclusive reform (i.e., Fisher et al., 2000; Mayrowetz & Weinstein, 1999), established inclusive schools (e.g., Hoppey & McLeskey, 2013; McLeskey et al., 2014), and educators’ experiences working in different inclusive schools (e.g., An & Meaney, 2015; DeMatthews et al., 2021).

Table 1 provides an abbreviated purpose/research question for each study and the methods used. All studies included qualitative methods, with some using mixed methods (e.g., Causton-Theoharis et al., 2011; Salisbury & McGregor, 2002). However, 17 (61%) were case studies, including case studies of individual schools (*n* = 10), multiple case studies that included two or more schools (*n* = 6), and one case study of a district. As Table 1 shows, all studies relied on interview data, 15 studies collected observation field notes, and 5 used surveys.

**Table 1.** Summary of Study Purposes and Methods.

Citation	Study purpose/questions in brief	Participants				Method				Data collected		
		Principals	SETs	GETs	Others	Case study			Other Qual.	Interviews	Observations	Surveys
						School	Schools	District				
Able et al., 2015 ^a^	Identify the needs of educators who teach students with autism spectrum disorder (ASD) in inclusive classrooms		X	X					X	X		
An & Meaney, 2015 ^a^	Describe four physical education (PE) teachers’ practices of including students with disabilities in PE classes and their experiences with Individualized Education Programs (IEPs)			X					X	X	X	
Causton-Theoharis et al., 2011 ^b^	Identify the accomplishments and barriers of a partnership designed to support inclusion	X	X	X		X				X		X
DeMatthews, 2015a ^b^	Examine how leadership was distributed for a more inclusive school	X				X				X	X	
DeMatthews, 2015b ^b^	Examine principal leadership in a low-income urban public school as they became more inclusive	X	X	X		X				X	X	
DeMatthews, 2018 Black boys^b^	Investigate the experiences of two principals working in a segregated school as it became more inclusive.	X					X			X	X	
DeMatthews, 2021 ^b^	Explore leadership behaviors in supporting effective inclusion	X	X	X	X		X			X	X	X
DeMatthews & Mawhinney, 2014 ^b^	A study of two principals in a high-poverty district during inclusion	X					X			X	X	
DeMatthews et al., 2021 ^a^	Examine six school principals’ thoughts as they sought to be more inclusive	X							X	X		X
Downing et al., 2004 ^b^	Examine first year of a charter school startup designed to be fully inclusive	X	X	X	X	X				X		
Downing et al., 2007^a^	Examine stakeholders’ perceptions when inclusion is expected		X	X	X				X	X		
Finke et al., 2009 ^a^	Examine perspectives of five GETs who included students with ASD			X					X	X		
Fisher et al., 2000 ^b,c^	Describe changes of an inclusive school and what sustained it		X	X		X				X	X	
Hoppey & McLeskey, 2013 ^b^	Examine how a principal provided support for inclusion in an era of high-stakes accountability	X				X				X	X	
Hoppey et al., 2018 ^b,c^	Describe how an inclusive philosophy was developed, implemented, and sustained	X	X	X			X			X	X	
Kangas, 2017 ^a^	Understand the conditions that influence the collaboration of GETs and specialists	X	X	X	X		X			X	X	
Kent-Walsh & Light, 2003 ^a^	Described GETs’ experiences with the inclusion of students who use augmentative and alternative communication (AAC)			X					X	X		
Keyes et al., 1999 ^b^	Identify principal leadership behaviors that strengthen teachers’ efforts for inclusion	X	X	X	X	X				X	X	
Ko & Boswell, 2013 ^a^	Describe PE teachers’ experiences teaching students with disabilities and their needs for support			X					X	X		
LaMaster et al., 1998 ^a^	Understand six PE teachers’ perspectives about inclusion			X					X	X		X
Lohrmann & Bambara, 2006 ^a^	Describe 14 GETs’ experiences including students with developmental disabilities and with challenging behaviors			X					X	X		
Mamlin, 1999 ^b^	Discover what inclusion looked like and the meaning it had for participants within a school and partnership	X	X	X	X	X				X	X	
Mayrowetz & Weinstein, 1999 ^b,c^	Examine leadership in a district known for high-quality inclusion practices	X	X	X	X			X		X	X	
McLeskey et al., 2014 ^d^	Identify factors contributing to the success of a highly inclusive school	X	X	X		X				X	X	
Salisbury, 2006 ^b^	Examine perspectives of eight elementary principals in developing inclusive schools	X							X	X		
Salisbury & McGregor, 2002 ^b^	Understand school context and leadership practices of principals who articulate an inclusive agenda	X	X	X					X	X		X
Theoharis et al., 2016 ^b^	Describe inclusive schoolwide reform, what changes result, and the role of leadership	X	X	X			X			X	X	
Waldron et al., 2011 ^d^	Examine the role of the principal in a highly successful elementary school	X	X	X		X				X		

*Note*. SETS = special education teachers; GETs = general education teachers.

a Teachers’ and principals’ experiences in different schools. ^b^Inclusive school reform in one or more schools. ^c^Sustainability of inclusive reform. ^d^Established inclusive school.

Some of the studies focused on a single group of educators. Five studies focused primarily on GETs (Able et al., 2015; Finke et al., 2009; Fisher et al., 2000; Kent-Walsh & Light, 2003; Lohrmann & Bambara, 2006), three focused on physical education (PE) teachers (An & Meaney, 2015; Ko & Boswell, 2013; LaMaster et al., 1998), six focused on principals (DeMatthews & Mawhinney, 2014; DeMatthews, 2015a; DeMatthews, 2018; DeMatthews et al., 2021; Hoppey & McLeskey, 2013; Salisbury, 2006), and others included a combination of educators (see Table 1).

Most studies focused on inclusion for all students identified with disabilities in the school(s) (e.g., DeMatthews, 2015b; Keyes et al., 1999; McLeskey et al., 2014), but several focused on students with: (a) autism spectrum disorder (ASD; Able et al., 2015; Finke et al., 2009), (b) emotional/behavioral disorders (DeMatthews, 2018), and (c) developmental disabilities (Lohrmann & Bambara, 2006). Two studies focused on students who used augmentative and alternative communication (AAC) devices (Finke et al., 2009; Kent-Walsh & Light, 2003). A few studies explicitly identified the inclusion of other groups, such as English Language Learners (ELLs) and Title I students (e.g., Fisher et al., 2000; Kangas, 2017); others focused on students with disabilities and documented the number of students by disability category including the number of students with LD, but emphasized the purpose of inclusion was to address the needs of all students in the school (e.g., Causton-Theoharis et al., 2011; McLeskey et al., 2014).

Of note, we did not identify a single study focused on educators’ working conditions when serving students with LD in inclusive settings. This omission is surprising, as students with LD comprise 33% of all students with disabilities, the highest prevalence category of eligibility (U.S. Department of Education, 2024). Accordingly, it is reasonable that students with LD would have been a part of these inclusive schools and participated in many of the classrooms included in this study. For example, over half of the studies focused on principals who led inclusive schools and only one focused on students with a specific exceptionality. DeMatthews (2015b) also reported that 54% of students with disabilities in their case study of an inclusive school were identified as having LDs. Thus, the omission of any discussion about students with LD in these inclusive settings is particularly glaring.

Across studies, participants supplied varied meanings and definitions of inclusion. Some described inclusion as having a basis in the LRE (e.g., DeMatthews & Mawhinney, 2014), as a right (Fisher et al., 2000), as the valuing of diversity and the creation of communities of belonging (Causton-Theoharis et al., 2011), and creating classrooms where all students’ needs can be met (e.g., McLeskey et al., 2014). One principal defined inclusion as students having:

access to the general education curriculum and all the rights and experiences of their peers. It’s not about shutting anyone out. . . . It’s making accommodations and doing whatever is needed to support a student. It’s also a belief that all children can learn and be successful. (DeMatthews, 2015b, p. 12)

Other principals acknowledged students may need alternatives to general education classes, with one stating:

Inclusion is about placing all students in their least restrictive environment . . . [and] almost every student should be included in the general education class . . . for some kids, at a particular point in their life, the general education class isn’t what’s best. . . . Our job is to make sure we get each kid to a place where they can thrive and be successful in inclusion. (DeMatthews & Mawhinney, 2014, p. 865)

### Working Conditions in Inclusive Schools

Our findings address educators’ perspectives on (a) their responsibilities in inclusive settings, and (b) resources on which they relied. We identified participants’ roles when possible, using generic terms (administrator, educator) if their role was not clear. Because principals’ roles are different and also help to shape working conditions within the school, we first describe findings for principals, and then findings for other educators.

#### Principal Responsibilities and Resources

##### Responsibilities

Across the principal studies, major responsibilities for inclusion encompassed (a) communicating an inclusive vision and celebrating successes; (b) enhancing capacity; (c) monitoring and adjusting implementation efforts; and (d) managing conflicts and problems. These responsibilities were often intertwined with and shaped by school context and how long the school was working to be inclusive. For example, a principal in a high-performing, well-established inclusive school with district support spent less time communicating a vision but continued to focus on building teacher capacity (Hoppey et al., 2018). By contrast, a principal in a recently segregated school in a low-performing district spent more time communicating a vision and managing emerging conflict (DeMatthews & Mawhinney, 2014).

###### Communicating Vision and Celebrating Successes

The most reported roles and responsibilities among principals were related to communicating an inclusive school vision and celebrating successes to build momentum (e.g., Waldron et al., 2011). For some principals, communicating a vision included demonstrating concern for the experiences and outcomes of students with disabilities in segregated settings (e.g., DeMatthews, 2015b). Many principals cultivated common language for inclusion, shared examples of student successes to build buy in, and used those successes to press forward in further dismantling segregated settings (e.g., Salisbury & McGregor, 2002). Some principals also shared this inclusive vision and successes with families, district leaders, and university partners to bolster partnerships and support (e.g., Causton-Theoharis et al., 2011).

###### Enhancing Individual and Team Capacities

Principals often reported responsibility for enhancing educators’ capacity, both individually and collectively (e.g., DeMatthews, 2015a, 2015b, 2021; Theoharis et al., 2016). Several principals—particularly those in lower performing schools and those more recently implementing inclusion—felt responsible for recruiting, hiring, and retaining high-quality teachers who valued inclusion and teamwork (e.g., DeMatthews, 2015a, 2015b; Keyes et al., 1999). They sought to provide high-quality professional development (PD), often including both individualized support for struggling teachers and schoolwide PD on topics, such as co-teaching and effective professional learning community structures and practices (e.g., Downing et al., 2004). Principals took ownership for this work. One principal noted, “I believe in creating experts in your building and encouraging them to coach others” (McLeskey et al., 2014, p. 65).

Principals also felt responsible for enhancing grade-level teams, co-teacher teams, and school improvement teams. Many reported efforts to support teacher leaders, especially as these individuals managed teacher teams and individualized education program (IEP) meetings (e.g., DeMatthews et al., 2021). Enhancing team capacity required principals to think carefully about time use and regularly meet with teachers (e.g., Downing et al., 2004). Several principals discussed mentoring teacher leaders, providing them feedback on their facilitation, and empowering them in planning meetings (e.g., Hoppey & McLeskey, 2013). Others noted team members needed to express frustrations or offer critique (e.g., Keyes et al., 1999). As one principal stated, building capacity was interconnected:

Hiring is important for obvious reasons, but I spend an equal amount of time on induction. I want you to know that our school, we really care about all students, we work together, and we do not have excuses. I think the biggest part of training teachers is not PD, it’s the signals you send as principal about what is valued and what is not accepted on your campus. (DeMatthews, 2021, p. 12)

###### Monitoring and Adjusting Implementation Efforts

Principals felt responsible and played roles in monitoring inclusion and engaging in shared and data-informed decision-making processes to make ongoing adjustments (e.g., Waldron et al., 2011). In schools that more recently adopted inclusion, principals reported working closely with educators to understand the context, collect data, and revise plans, procedures, and policies (e.g., budget, master schedule, staffing models, service delivery models, teacher caseloads, and discipline policies (e.g., Mayrowetz & Weinstein, 1999; McLeskey et al., 2014; Waldron et al., 2011). One principal noted that inclusion “takes incredible coordinating, incredible planning. We meet, we meet, we meet . . . because you cannot coordinate all these people and services without meeting” (Salisbury, 2006, p. 77).

The role of monitoring data was strongly emphasized. As one principal noted, “How can I have conversations with teachers about their students, how they’re progressing, how well they’re teaching without individual data about students” (Waldron et al., 2011, p. 57). These data points, coupled with shared decision-making approaches, enabled principals to identify effective policies and structures to adjust. A principal described this as “lubricating the human machinery” so teachers could be effective (Hoppey & McLeskey, 2013, p. 252). Relatedly, several studies described how principals supported used tiered supports for including students with disabilities (e.g., McLeskey et al., 2014; Waldron et al., 2011). For example, Hoppey et al. (2018) described how Response to Intervention (RTI) supported inclusion in two schools they studied, as it provided a “common language” about assessment and instruction (e.g., progress monitoring, universal screening, research-based interventions), supported teachers’ “meaningful conservations about curriculum” and the monitoring of student progress (p. 35).

###### Managing Emerging Conflicts and Problems

Several studies described the uncertainty of school leadership and emerging problems that were a part of inclusive schools regardless of context or implementation status (e.g., Salisbury, 2006). Principals reported feeling responsible for problem-solving or facilitating responses to conflicts, such as due process complaints, serious behavior problems, or budget cuts (e.g., Downing et al., 2004). In many cases, inclusion was working well for most students, but some struggled significantly, requiring regular meetings and efforts to maintain inclusion or adjust (e.g., DeMatthews, 2018). Principals also reported a proactive aspect of managing emerging problems—buffering teachers and staff from external pressures so they could do their jobs without worry (e.g., high-stakes accountability, bureaucracy; Hoppey & McLeskey, 2013; Mayrowetz & Weinstein, 1999). Parents of students with and without disabilities at times expressed concerns with the school or teacher, and principals felt compelled to intervene (DeMatthews & Mawhinney, 2014; Mayrowetz & Weinstein, 1999). One principal’s comments were illustrative of the role of managing conflict:

A principal’s job is never done. Probably the toughest thing about the job is you cannot schedule for it. I always have my day scheduled. I keep a journal. But I can flip through it and tell you I rarely get through everything because things always come up. A board member wants to visit, a parent is upset, there is some sort of emergency meeting, whatever, there are just always things. (DeMatthews, 2021, p. 15)

##### Resources

Principals sit in a unique organizational position between the district and the classroom and between the school and community. As such, principal working conditions are complex and intertwined at the school, district, and community levels. For example, a district may support inclusion and help foster a partnership that brings important resources to the school, yet the principal might still confront significant teacher resistance to inclusion given the district has not prioritized inclusion or offered teacher trainings (e.g., DeMatthews & Mawhinney, 2014; Theoharis et al., 2016). In what follows, we outline findings on district and school resources based on our review of this smaller body and more limited body of literature.

###### District Resources

Most studies provided insight into how districts supported or constrained principals’ efforts to lead inclusive schools (e.g., Causton-Theoharis et al., 2011). Districts often both supported and constrained principals’ efforts, to varying degrees. A primary way districts supported principals was through having district leaders who advocated for inclusion, supported innovation, and risk-taking, and provided adequate resources and partnerships (e.g., Mayrowetz & Weinstein, 1999; McLeskey et al., 2014). For example, one principal noted that her district always supported her: “When I was hired, the superintendent said, ‘We want risk takers, not caretakers.’ We are expected to have new thoughts and ideas” (Salisbury & McGregor, 2002, p. 266). Some districts also provided teachers with PD to prepare them for inclusion, thereby reducing principals’ load for capacity building (Hoppey et al., 2018).

For many principals, district resources were lacking, affecting their ability to create and sustain inclusive schools. Budget cuts, bureaucratic demands, limited or ineffective PD, and a set of inclusion-related disincentives appeared as constraints across several studies (e.g., DeMatthews, 2015a). In one study, principals used terms such as “juggling,” “spreading thin,” and “insufficient” to describe the lack of resources available to support their efforts to promote inclusion (DeMatthews et al., 2021). One principal noted, “We have so many kids and so little staff . . . it’s a constant process of adjusting” (DeMatthews et al., 2021, p. 32). Even in a more affluent school, the district cut one of the two SET positions amid the school’s effort to promote inclusion, requiring the principal and others to help with administrative and legal demands to protect the sole SETs’ time (DeMatthews, 2015a). Principals frequently reported challenges with resources, especially concerning SET caseloads and workload burdens associated with IEP development and related paperwork. As a result, some principals were frustrated by having to overwork teachers, which could contribute to additional teacher resistance and frustration they would then have to manage (e.g., DeMatthews, 2015b; DeMatthews & Mawhinney, 2014).

Current and prior district policies and practices also constrained principals’ efforts. DeMatthews et al. (2021) and Mamlin (1999) reported how “legacies of segregation,” or a “culture of segregation” made inclusive leadership extremely difficult for principals. For example, a parent of a child with a disability was fighting with the principal and was strongly against an inclusive placement because she felt the school was removing hard-fought services that were helping her child (DeMatthews, 2018). The principal understood why the parent felt this way given the district’s track record of denying quality services and viewed the parent’s behavior as a burden she had to bear, as she took additional time to build trust with the parent—effort that could have been placed elsewhere had the district had a better track record. Other studies described how well-documented district failures made building trust with families difficult and caused additional resistance to inclusion (e.g., DeMatthews & Mawhinney, 2014).

Several studies reported that schools with self-contained programs were “penalized” for moving students with disabilities into general education classes. For example, when principals worked to move students in self-contained programs into inclusive classrooms, some districts would simply add more students from other schools into the self-contained program where a seat was now available (DeMatthews & Mawhinney, 2014; Hoppey & McLeskey, 2013). Principals also noted several other district concerns, such as inflexible policies such as requiring principals to attend every IEP meeting—a significant time investment (Salisbury, 2006). A broad array of district demands and bureaucratic red tape were also perceived as challenges. One principal said:

It’s hard to lead if you cannot always be present when your faculty or staff need you . . . I’ve been called out of the building or had to finish a report that was given to us last minute. . . . Just being a principal can be a distraction from your priorities. (DeMatthews, 2021, p. 15)

Perhaps due to a lack of trust or communication, at least one principal feared that inclusion would ultimately lead to budget cuts if students with disabilities were receiving fewer pull-out services (Hoppey et al., 2018). Other studies noted that districts placed significant pressure on test scores associated with high-stakes accountability (e.g., Hoppey & McLeskey, 2013). Another principal described how their proximity to charter schools created a serious challenge when those schools’ expelled students with disabilities after an enrollment audit, meaning their public school would need to enroll more students without more resources (DeMatthews & Mawhinney, 2014).

##### School Supports

Most studies included principal perspectives on supports at the campus level. Principals primarily identified educator resistance as a demand. They also identified prior standard operation procedures and expectations as a demand if the school was transitioning to becoming more inclusive. Both demands were tied to the extent that teachers and staff were prepared to implement inclusion, which we have included in each of the subsequent sections.

###### Educator/Staff/Parent Support and Resistance

The most common school-level support or constraint reported by principals was support for or resistance to inclusion (e.g., Mamlin, 1999). Across most studies, principals frequently found a mixture of support and resistance. Some principals reported that teachers petitioned for inclusion and provided support by making time investments in planning, developing new skills, and helping communicate the importance of inclusion to others (Causton-Theoharis et al., 2011; DeMatthews et al., 2021). One principal recalled teachers coming to her when she was first hired, saying, “We need to make a change because some of kids in SPED are failing” (DeMatthews, 2021, p. 13). Causton-Theoharis et al. (2011) examined a university–school district partnership that restructured the school for inclusion. The principal noted that inclusion was “something the staff wants to do” and the partnership was “exciting and rejuvenating” (p. 199). Survey results verified the principal’s perspective as just 4 out of 44 staff members surveyed indicated a lack of support for inclusion and they had not been active in meetings or the improvement process. In an affluent school, the principal had some staff who were less engaged, but she found significant support from one SET who was supportive of inclusion and who was an effective teacher advocate among her peers (DeMatthews, 2015a).

Many teachers were not resistant to inclusion in principle or not initially, but instead resisted under certain conditions—particularly when they viewed inclusion as an added burden. Principals in several studies reported teacher resistance only after significant changes were made, such as shifting resource allocations, caseloads, or the ways teachers were expected to plan lessons and approach classroom management (e.g., Salisbury, 2006). Relatedly, some principals reported teacher resistance for certain disability groups, such as students with emotional and behavioral disabilities (DeMatthews, 2018). Other principals reported passive resistance. As the principal of a high-performing school noted, “It’s hard to make change when you are in the high 90s (student achievement levels). The teachers believed they had evidence that they were great teachers, and this made change difficult” (DeMatthews, 2015a, p. 1015).

Other principals confronted far greater teacher resistance, especially early in the planning and implementation process (Mayrowetz & Weinstein, 1999). One principal reported how a teacher publicly challenged and disrespected her during a staff meeting and how others refused to adhere to schoolwide discipline policies for students with disabilities (DeMatthews, 2015b). Such resistance was detrimental to her well-being and overall effectiveness: “It’s upsetting. It didn’t feel good, it made me look weak” (p. 155). Other forms of resistance reported by this principal and others were covert and indirect, which included coming unprepared to important meetings, coaching parents to push for their child to be moved into self-contained programs or speaking ill of inclusion and the principal to colleagues (DeMatthews, 2015b). Another principal captured how resistance was disheartening: “What I hate to hear is a teacher saying that’s not my student” in reference to a child with a disability (DeMatthews et al., 2021, p. 32).

In some cases, parents and communities were resistant to inclusion. Some principals reported resistance from parents of children with disabilities. These parents were not enthusiastic about inclusion and feared their child would lose services and be bullied in general education classes (DeMatthews & Mawhinney, 2014). In the same study, a principal in a gentrifying community viewed parents as resistant, particularly after expressing concern about Black children with disabilities being bused into the school as part of a self-contained program the principal was working to dismantle. However, parents could also be a great support to principals, particularly when they supported initiatives, helped raise funds, and were open-minded about how their child was served (Downing et al., 2004). Likewise, community organizations and non-profits were reported to provide support for inclusion by bolstering the budget and assisting in planning and preparation (Hoppey & McLeskey, 2013).

###### Standard Operating Procedures and Expectations

Many principals reported preexisting standard operating procedures and expectations supported or constrained their work (e.g., Mayrowetz & Weinstein, 1999). Some schools had existing procedures and expectations that lent themselves to collaborative work central to creating inclusive schools while others lacked such procedures and expectations. For example, some schools had a collaborative and collegial culture, high-quality PD, and a longstanding emphasis on using data and engaging in continuous improvement (DeMatthews, 2015b). In one case, the principal already had access to teacher leaders who facilitated grade-level teams and were able to use data to inform decisions. These preconditions enabled inclusion to be adopted with less initial individual and group PD and support (DeMatthews, 2015b). However, regardless of school context, inclusion required a shift of operating procedures and expectations that created challenges for principals.

Even in a school with a collaborative culture and with some prior conditions that might support inclusion, one principal described how, prior to her arrival, the school “conformed to a rigid schedule that included pull-out sessions for specialized instruction and related services” and that many teachers did not differentiate instruction (DeMatthews, 2015a, p. 1014). In another study, a principal described how prior expectations created constraints for inclusion:

Because those (SpEd) teachers were not comfortable just walking into the classroom . . . they were used to sitting with 3 or 4 kids—and this was a real adjustment for them to be in front of 28 kids and responsible for part of the lessons. (Salisbury, 2006, p. 78)

Some schools were even less collaborative, and data use for continuous improvements was not part of teachers’ expectations for their practice. Other schools did not have natural proportions of students with disabilities in classes which created additional challenges for principals seeking to shift students with disabilities into new classrooms (Salisbury, 2006; Theoharis et al., 2016).

The lack of preconditions to support inclusion meant principals needed to spend more time and energy on building consensus. A principal of an elementary school in Texas who was working to make her school more inclusive noted, “Before you can get people on board [for inclusion], you have to make sure the conditions are in place for people to listen and work together” (DeMatthews, 2021, p. 12). Another principal in an urban district noted that the prior principal in her school was known as a “disciplinarian” who “suspended students constantly” (DeMatthews, 2018, p. 414). From the principal’s perspective, this history created teacher expectations that the principal should suspend students with disabilities for behavioral infractions rather than shifting attention to how they can improve classroom management. Another principal in a school without a culture of teacher collaboration or a track record with inclusion noted, “Right now, we are more concerned about adult needs than student needs and that needs to change” (DeMatthews, 2015b).

#### Educators’ Responsibilities and Resources

##### Responsibilities

Most of these studies focused on GETs’ responsibilities, with relatively few examples from SETs, paraeducators, and related services personnel. GETs’ responsibilities changed as they worked to meet the needs of students with disabilities in inclusive classrooms (e.g., Fisher et al., 2000). One SET commented her transition to an inclusive setting was a “hard shift” (Hoppey et al., 2018, p. 30) while a GET commented, “teachers were taking on . . . very different roles and responsibilities than we had been used to” (Causton-Theoharis et al., 2011, p. 199). Thus, educators’ responsibilities were, at least in part, redefined as students were included in inclusive classrooms. These findings consider primarily GETs responsibilities as they (a) learned about and taught students with disabilities, (b) facilitated a culture of inclusion and supportive peer relationships, (c) addressed challenging behavior, and (d) collaborated with SETs, paraeducators, specialists, and families.

###### Learning About and Teaching Students With Disabilities

Many GETs felt unprepared to teach students with disabilities (e.g., Able et al., 2015; Ko & Boswell, 2013; Lohrmann & Bambara, 2006), and they described their efforts to understand each student and to learn about effective strategies to meet each student’s needs. In a study of GETs who instructed students with autism using AAC devices, an educator highlighted the need for a comprehensive understanding of the student, saying,

For Joe to succeed, it is essential for us to grasp his personality and specific needs comprehensively. This understanding not only facilitates our support for Joe but also enhances the learning environment for all students in the classroom. (Finke et al., 2009, pp. 118–119)

In a study of four experienced PE teachers, a teacher said they needed to understand “what their deficiencies are”; while another focused on “students’ goals and abilities,” adding, “You have to get to know the students pretty well where they are at, what they can or can’t do” (An & Meaney, 2015, pp. 148–149). Some teachers described the importance of reviewing students’ IEPs and wanted to be involved in IEP meetings as an opportunity to interact with the team and provide input into the student’s plan (An & Meaney, 2015). However, some were not included in IEP meetings and felt “out of the loop” (Kent-Walsh & Light, 2003, p. 112). A PE teacher shared:

I really want to help them create good goals for this girl because I think that often time if the PE teachers doesn’t sit in or somebody else sits in, the goals are little bit weaker . . . and not specific enough and so I guess I see my role in the IEP process this year as bringing my knowledge to get to the right goals for the child. (An & Meaney, 2015, p. 153)

GETs’ responsibilities were often framed in terms of accommodating or modifying instruction for students with disabilities, as they sought to provide students with disabilities with access to general education curricular content. Downing et al. (2004) examined the startup of an inclusive charter school and described how, while some students needed only simple accommodations, such as enlarged print, others required rewriting material for a lower grade level. In Downing and Peckham-Hardin’s (2007) qualitative case study in an inclusive charter school, teachers described varied modifications, such as simplifying content, using pictures paired with print, rephrasing questions, and providing alternatives to writing, among other examples. They also identified the importance of “structure and consistency, as well as providing frequent opportunities to review and practice. . . to enhance mastery of content” (Downing & Peckham-Hardin, 2007, p. 23).

PE teachers also provided access to physical activities by modifying instruction, allowing some students to dribble with two hands or use balls of varied sizes and weights (An & Meaney, 2015), and changing distances between students and targets (Ko & Boswell, 2013). PE teachers often experimented to find accommodations or modifications as Ben, a teacher explained:

For me personally, it’s more of a trial-and-error process. . . . seeing what they [students with disabilities] can do, then tailoring the lesson to them so they’re included. I put myself in their shoes and say, “Well I know I can’t push that ball to the goal . . .so what could I do? So, let’s try this bucket.” (Ko & Boswell, 2013, p. 233)

GETs also supported students by incorporating assistive technology, such as adapted chairs (Downing & Peckham-Hardin, 2007) and AAC devices (Finke et al., 2009; Kent-Walsh & Light, 2003). Importantly, some GETs pointed out how they adjusted instruction for *all* students, not just students with disabilities (Downing & Peckham-Hardin, 2007).

Although GETs’ descriptions of inclusion were often positive, they also described challenging responsibilities. A PE teacher described struggling to teach “two lessons at the same time . . . because you are constantly trying to keep one student busy” (LaMaster et al., 1998, p. 75). In Finke et al.’s (2009) qualitative study of GETs including students with ASD who used AAC devices, a GET noted the demands of planning for an inclusive class:

It’s hard to plan and create these lessons as well as create modifications needed to make the lesson successful for children needing additional assistance. Teachers work so hard as it is and so much time is put into one work day. (p. 115)

Teachers also described challenges during instruction. For example, GETs commented they had insufficient time to learn about students’ AAC systems, which limited their ability to support their use of these devices (Kent-Walsh & Light, 2003). They also needed to help students troubleshoot when devices did not work and figure out how to motivate students to use them (Kent-Walsh & Light, 2003). Other GETs described feeling like they had to triage their attention, as they could not give all students what they felt they needed within a single class period (e.g., LaMaster et al., 1998). In Lohrmann and Bambara’s (2006) study, a GET who initially felt confident he could support a student felt that limited time in the school day left him feeling that he was not serving all students well: “You want to do something to help that child, but you still have all these other ones you need to be working with too. . .the time isn’t there” (p. 164).

###### Facilitating a Culture of Inclusion and Peer Relationships

GETs demonstrated concern for and a responsibility for students with disabilities that extended beyond academic progress to encourage acceptance of all students (e.g., Finke et al., 2009) and to support the development of social skills (e.g., Able et al., 2015). Downing et al. (2004) reflected on how the culture of inclusion in the school transcended disability and applied to students with other marginalized identities: “It’s school-wide, the level of acceptance. And it’s not just kids with disabilities . . .. Even the heavy-set kids, you know? And we model that acceptance and . . . . the kids have . . . embraced it” (p. 17). Another GET tried to normalize students’ support needs:

They [all students] are taught from the start that all people learn differently and at our school we help them figure out how they learn best. They understand that some students may need more support than others. All of the children in class get along and are very supportive of each other. They work together and help one another learn best. (Finke et al., 2009, p. 119)

Likewise, in Lohrmann and Bambara’s (2006) study, teachers described “demystify[ing]” the disability by explaining the “student’s disability and needs” (p. 168), although in another study, a teacher wondered if it was appropriate to share information about a student with a disability due to confidentiality (Able et al., 2015).

Teachers also considered social interactions between students with and without disabilities. Lohrmann and Bambara (2006) found that 13 of the 14 GETs they interviewed assumed responsibility for facilitating relationships between students with developmental disabilities and those without, as it is “part of why the student was being included. . .” (p. 168). GETs encouraged peer buddies, modeled positive interactions, and interpreted when peers did not understand a students’ speech or actions (Lohrmann & Bambara, 2006). Finke et al. (2009) also described that students:

will often work together and include the child with autism; they may share ideas, or sometimes even discuss things off topic as they are working. I find this allows the students with autism to build friendships and relationships in the classroom and not only out on the yard. (Finke et al., 2009, p. 113)

GETs expressed that supporting these peer interactions was important given that students with disabilities were sometimes positioned as less-valuable peers in interactions with classmates (e.g., Downing & Peckham-Hardin, 2007). One teacher noted that students without disabilities “demonstrated an obligation” to socialize with students with disabilities, “rather than having a desire to do so” (Kent-Walsh & Light, 2003, p. 111).

###### Addressing Student Behavior

GETs also spoke about their responsibility to create a positive, productive learning environment for all students (e.g., Lohrmann & Bambara, 2006) and expressed concern when students with challenging behaviors disrupted their classrooms. Sometimes, they expressed concerns about behaviors that they felt were not harmful, but that made it hard for students to concentrate (e.g., LaMaster et al., 1998). For example, GETs felt inclusive settings were sometimes overwhelming for their students with ASD and described how their verbal and behavioral outbursts affected teachers’ ability to teach and all students’ ability to focus. As a GET stated, “For the other students in the class . . . the classroom can become very noisy with students of all abilities and my typically developing students have at times told me they were having a hard time hearing me or concentrating” (Finke et al., 2009, p. 115).

Other times, when students engaged in unsafe behaviors, teachers worried about the impacts of those behaviors on both their instruction and other students’ sense of safety. One GET said that a student was “frightening when he began throwing things,” while another said, “It’s a scary situation when you don’t feel safe, and you want to feel safe for your children” (Lohrmann & Bambara, 2006, p. 164). Downing et al. (2004) described student behavior as a continuing challenge in the first year of an inclusive charter school, which included “screaming, running off, kicking, and refusing to work” (p. 20). DeMatthews (2015b) described the struggles teachers experienced teaching students with emotional disabilities in an urban, high-poverty school. As the school included more students with disabilities in general education, more teachers struggled with behavior, with “20 to 25 kids . . . in trouble every day” (p. 14). A discipline committee was formed to identify students needing behavioral support, connect students with interventions, and develop an intervention matrix. Although teachers viewed the committee as moderately successful, concerns about behavior continued (DeMatthews et al., 2015b).

Teachers sometimes removed students from the classroom when they engaged in “disruptive behavior” (Downing & Peckham-Hardin, 2007), a solution teachers were concerned about. One teacher commented on a student missing a lesson:

It’s hard to feel like they [students with behavioral challenges] are learning on a daily basis. . . . And also, they spend so much time out of the classroom because of need-based breaks. . . . I’ll have this great lesson and then [student] will tantrum about something at times and it’s just so frustrating because I’ll be heart-broken that he didn’t participate because I had it in my mind that he would just love what we were doing. (Downing & Peckham-Hardin, 2007, p. 21)

In another school, DeMatthews (2015b) described that while some students with emotional disabilities improved, one student who bullied, hit, and teased other students was returned to his prior placement as he “. . . wasn’t ready” and “his grades suffered” (p. 19), which disappointed the teachers who worked hard to include him.

Although supporting students’ behavior was a challenging responsibility for GETs and schools created teams to address behavior (e.g., Keyes et al., 1999), there were no descriptions of evidence-based practices to address behavior. Lohrmann and Bambara (2006) commented that “None of the teachers were able to describe a systematic process for arriving at strategies other than brainstorming and trial-and-error” (p. 168) and only one teacher was familiar with Positive Behavioral Supports as a parent shared this approach.

###### Collaborating With Educators and Families

Collaboration is an expected activity in addressing the needs of students with disabilities. Educators viewed collaboration as an important responsibility and necessary for planning to address the needs of students with disabilities. A GET commented, “I think it is really important to get together on a regular basis to conference with the speech teachers, the school psychologist, the IEP teacher or anyone who happens to be working with this child” (Lohrmann & Bambara, 2006, p. 165).

Several schools also created organizational arrangements to support co-teaching. Causton-Theoharis et al. (2011) described how each SET along with two paraeducators were assigned to two general education classrooms, providing opportunities for GETs, SETs, and paraeducators to work together regularly as a team, which also reduced the number of colleagues that each teacher needed to collaborate with on a regular basis. Lohrmann and Bambara (2006) described how teachers were resentful from the additional inclusive responsibilities until “a collaboration structure (i.e., early release day once a month for meeting time) was put in place and training for all staff occurred . . .” (p. 165).

Teachers also addressed what was accomplished during collaboration, such as setting goals for students (Hoppey et al., 2018), discussing student problems and brainstorming solutions (Lohrmann & Bambara, 2006), and planning for co-teaching (e.g., Downing & Peckham-Hardin, 2007; Hoppey et al., 2018). As a teacher stated,

The more we can get together to plan, which we do often, it’s better. . . . The most important thing is to plan with the co-teacher, then we know what we’re going to do, and know whose group is going to do what. (McLeskey et al., 2014, p. 66)

Several studies referred to co-teaching, although their responsibilities were not described in detail (e.g., Fisher et al., 2000; Hoppey et al., 2018).

Fulfilling their collaborative responsibilities also required that team members sometimes had to navigate complex interpersonal dynamics. For example, Finke et al. (2009) reported difficulties working as a team, and a teacher commented, “Knowing our boundaries and expectations of the specialists’ jobs, regular educators’ jobs and responsibilities, and the special educators piece—is difficult to establish at times” (p. 116).

Although the effectiveness of collaboration was not typically addressed, Theoharis et al. (2016) described how some collaborations,

. . . resulted in less meaningful differentiation, accommodations, and modifications. A number of teams did not know how to use multiple adults in effective ways and oftentimes everyone in the special education staff looked more like teaching assistants. (Theoharis et al., 2016)

Collaboration with parents of students with disabilities was also an important responsibility, although GETs were not necessarily the primary point person for these interactions (An & Meaney, 2015). PE teachers generally expressed the importance of family communication and wanted more opportunities to work with families (e.g., An & Meaney, 2015). GETs likewise valued their work with families, yet sometimes felt overwhelmed by this responsibility. In Finke et al.’s (2009) study, a teacher shared how she realized that parents were “fighting for their children who cannot fight for themselves,” yet she noted the substantial demands of “some very demanding parents—whether they are demanding with their communication log expectations or services their child is/isn’t receiving” (p. 116). Teachers also worried about letting parents down. For example, one GET shared:

If we are having a class performance, the chances are slim that Joe will participate. I know this is at times difficult for his parents. They want to see their child fully included and it’s not that we aren’t trying. (Finke et al., 2009, p. 116)

GETs also spoke about their responsibilities to collaborate with families of children without disabilities, who were sometimes concerned about the effects of inclusion on their children (Finke et al., 2009). One GET shared that “teachers are confronted with the reactions of parents of ‘normal’ children who are afraid that their child will get less attention,” while another said, “At times I have heard . . . that parents feel students with special needs are given more support and attention during the day” (Finke et al., 2009, p. 116).

###### Dilemmas Teachers Experienced

In several studies, GETs had questions about the nature of their work with students with disabilities in their classes (e.g., what behaviors to ignore, how they should prioritize different needs) (e.g., Able et al., 2015; Kent-Walsh & Light, 2003). Although the authors of these studies did not delve deeply into these questions, they were evident in the findings. In Able et al.’s (2015) study of GETs teaching students with ASD, GETs described wondering what behaviors they were and were not responsible for addressing:

If a student with ASD is working diligently but has his feet on another desk and is making weird sounds, the important thing to me is that he is working. But I often wonder should that be the real focus, or should the focus be on where his feet are and the weird sounds he is making? (p. 51)

This GET was trying to understand the extent to which her responsibility included enforcing neurotypical behavioral norms versus ensuring students were engaged in academic content. Some GETs also questioned the value of accommodating students’ access to general education curricula versus providing students intensive intervention instruction in areas where they felt students could make more meaningful progress. For example, one upper elementary GET described making modifications so a student with a developmental disability could relate to content on the Revolutionary War “at a very basic level” (Kent-Walsh & Light, 2003, p. 114), but wondered whether this was the best use of the student’s time relative to reading instruction, which she felt would yield more benefit. Likewise, a PE teacher felt, “Some (inclusion) students do not benefit from spending minutes in a physical education class when there are other things. . .that would be more appropriate” (LaMaster et al., 1998, p. 74). By contrast, other GETs were concerned about students’ limited progress in the general education curriculum, and worried that they were not doing enough to support them (Lohrmann & Bambara, 2006). None of these studies delved into teachers’ questions or what they may have relied on to help resolve them.

##### Resources

Educators relied on or needed varied types of resources to fulfill these responsibilities, including social supports from principals, other teachers, paraeducators, related services personnel, peers without disabilities, and parents; logistical supports (e.g., time, schedules, resources, space); and informational resources (e.g., PD, IEPs, varied types of written or digital information, and college classes).

###### Social Resources

To fulfill their responsibilities, GETs spoke about collaboration as they relied on this social support from other GETs, SETs, adapted physical education (APE) teachers, related services personnel, paraeducators, and other students, while others noted the absence of such supports. Across studies, teachers’ reliance on, and need for these collaborative supports was the most consistent and powerful finding.

Collaboration took time and was a responsibility for all educators, but they clearly valued collaboration as a crucial resource supporting their work. As one GET said, “If you have a problem, a coworker will say, ‘Hey, I’ve got materials for that,’ or ‘I know a way that will work’” (Fisher et al., 2000, p. 224). In Downing et al.’s (2004) study of a new inclusive charter school, a paraeducator commented on the importance of the social support:

But we were always there to provide support for each other. I don’t know if that is a measure of the people who work here, the school environment or the fact that it’s small. I don’t know. But it feels very supportive, for the kids and the grown-ups. (p. 18)

GETs noted how collaboration supported their professional learning (e.g., Downing et al., 2004), as colleagues listened to them, offered comfort and guidance, and provided feedback (Lohrmann & Bambara, 2006). A speech-language pathologist supported a GET by investigating resources for a student (Kent-Walsh & Light, 2003), and a PE teacher valued the support of an APE specialist, stating “I was able to expand my knowledge and help the kids” (An & Meaney, 2015, p. 149).

Because collaboration was so important, educators consistently commented on how they struggled when important colleagues were not available (e.g., Kangas, 2017). This sometimes occurred when key specialists were working across multiple schools; for example, some PE teachers expressed concerns that APE teachers were not consistently available (Ko & Boswell, 2013). Kangas (2017) conducted a multicase study of two elementary schools focused on how GETs, SETs, and English Language (EL) teachers collaborated to serve EL students with disabilities. At one focal school, the EL teacher was working across four or five schools, limiting opportunities to co-plan. The EL teacher commented: “I think if I were just in one building, obviously, I’d be much more effective ‘cause I could see those teachers before and after school every day and at their specials and even grab each other in the hallway” (Kangas, 2017, p. 266).

Paraeducators were also crucial sources of support, assisting specific students with disabilities and helping teachers and other students in the classroom (Mayrowetz & Weinstein, 1999). All 14 GETs who taught students with developmental disabilities and challenging behavior felt that in-class assistance was necessary to address the needs of these students (Lohrmann & Bambara, 2006). A GET stated it would be “impossible” to include a student who used AAC without an aide (Kent-Walsh & Light, 2003, p. 116). Other GETs described how the paraeducator “handled everything so easily and was able to make ‘on-the-spot’ decisions about how to reinterpret an assignment” (Kent-Walsh & Light, 2003, p. 116). Another referred to a paraeducator’s patience, ability to communicate with the student, and her helpfulness in assisting others in the class (Mayrowetz & Weinstein, 1999).

By contrast, some GETs described challenges when paraeducators were absent, unskilled, or inconsistent. For example, one GET described how a student had to miss her class when a paraeducator was absent, while another described how the paraeducator working with a student was inconsistent, challenging their capacity to establish routines for his participation (Kent-Walsh & Light, 2003). In LaMaster et al.’s (1998) study, PE teachers expressed frustration that, when paraeducators were absent, they had difficulty giving students the support they needed; one PE teacher described how a student left the room without permission when a paraeducator was out, such that she had to pause instruction, leaving her class alone, to find the student. Without consistent, trained paraeducators, including students with disabilities was more challenging.

GETs also identified parents as a crucial source of support, especially when they provided important information. For example, in Mayrowetz and Weinstein’s (1999) study, a parent made a video to show GETs what their child was able to do at home. An and Meaney (2015) described a PE teacher who relied on an email from a student’s mom and grandmother, which provided information about the student’s disability: “It just has all different things we should know about it” (p. 149). Downing et al. (2004) described how parents were involved in all aspects of the school and directly supervised students and volunteered for numerous activities.

GETs often engaged students without disabilities to support students with disabilities during instruction. For example, GETs relied on peers to support students using AAC devices (Finke et al., 2009, p. 114). A PE teacher referenced the use of peer supports as “one of the most useful teaching strategies” and encouraged students without disabilities to assist; another relied on students when paraeducators were absent, thus the “responsibility for some inclusion students . . . was handed over to other children” (LaMaster et al., 1998, p. 75). Although GETs often saw peers as resources, one was concerned about the “unequal status” of students with disabilities, as peers were more often in helper roles versus friend roles; the GET described needing “to stop them and say, ‘no, she can do that’” (Kent-Walsh & Light, 2003, p. 111).

Although studies of administrators often refer to them as crucial supports for inclusive school reform (e.g., McLeskey et al., 2014), teachers only occasionally spoke about the importance of administrators’ support. In Lohrmann and Bambara’s (2006) study, teachers appreciated administrators as critical sources of support, providing examples of how administrators were willing to “jump in and help, back the teacher up, and make life a little bit easier,” (p. 166), set expectations for academics and curriculum, support PD, and in general be encouraging. In Finke et al.’s (2009) study, teachers shared how administrators ensured they had the material resources they needed, supported time for them to meet regularly as a team, and were engaged with students daily, “visiting the classroom and doing informal observations so that they were able to speak first hand during IEP meetings” (p. 118).

##### Logistical Resources

Logistical resources include time, facilities, and materials.

###### Time

Educators consistently reported that time was a critical resource for fulfilling their responsibilities, including time to learn, collaborate, and teach. Teachers often found it difficult to work with others to manage their students’ learning and behavioral needs. As one teacher shared:

I run from planning, to teach here, then there, sometimes I don’t get to see each student as much as I want and in between that I don’t eat lunch. And I’m still being asked to do more, I still have to rewrite IEPs, it’s relentless here. . . . What’s worse, sometimes I just feel like I’m not doing a good job. Am I a good teacher? I don’t know. (DeMatthews, 2015b, p. 13)

In another study, a paraeducator described inadequate time for providing modifications:

There is not the time to do a lot of better modifications. . . . Sometimes we pump out a bunch of generic modifications . . . . We modify to the lowest ability and sometimes that is kind of disappointing. (Downing & Peckham-Hardin, 2007, p. 23)

Although educators appreciated when principals articulated dedicated time to these responsibilities (e.g., Lohrmann & Bambara, 2006), teachers reported having insufficient time to collaborate, even when it was identified as a priority (Causton-Theoharis et al., 2011). For example, GETs reported needing more planning time to learn to use AAC devices and figure out how to include target students, but “they were not given extra time to address these demands” and another described using her planning time to learn more about the student’s AAC, requiring her to work “past my time” (Kent-Walsh & Light, 2003, p. 112). Another GET described how she still needed extra preparation despite her investment in learning to use a student’s AAC device: “I need time and although I’m really, really interested and it was a priority for me, it was another layer that I had to do to prepare” (Kent-Walsh & Light, 2003, p. 112).

In Causton-Theoharis et al.’s (2011) study, most educators in one school viewed collaboration as a success and they appreciated the support that it provided, yet 40% of teachers surveyed indicated they needed more time to collaborate, while 70% of paraeducators reported needing more time to work with teachers. A paraeducator commented:

We have lessons to carry out with students that we may not be familiar with. It is very difficult to sit down with a group of students and read the plans for the first time and carry out the lesson. (Causton-Theoharis et al., 2011 p. 201)

In Kangas’s (2017) multicase study of how special education and EL services were coordinated in two elementary schools, an SET did not have common planning time with GETs, so she adapted instruction “on the spot” for a child with autism whose first language was Arabic:

They [general education teachers] plan their lessons. We don’t really plan their lessons because they’re planning it based off the curriculum. So, we won’t per se plan them differently than they are, but we do adapt their lessons. So . . . I mean, you see many times I’ll pull them [the students] into the back or pull them, so there’s adapting on the spot. (p. 266)

This study revealed how the lack of collaborative time and support limited the extent to which EL and special education services were integrated. The school had a policy that special education took precedence over EL services, and EL teachers were spread across multiple schools; as a result, only one EL teacher reported being able to co-plan with both GET and SET colleagues. This compartmentalized approach to meeting students’ needs prioritized their disability over their language support needs, rather than considering both simultaneously.

In a handful of studies, teachers had dedicated time for collaboration, either because their principals made time in the schedule (Finke et al., 2009), hired substitute teachers so they could attend weekly collaboration meetings (Kent- Walsh & Light, 2003), or provided an early release day for staff training on collaboration (Lohrmann & Bambara, 2006). One paraeducator appreciated daily debriefing sessions with the teacher stating, “I would . . . talk with [the teacher] about how the day had gone. Just about a six minute exchange. That’s important for the flow of things” (Mayrowetz & Weinstein, 1999, p. 440). Yet, teachers often reported that the time they had was insufficient; for example, four of the five GETs in Finke et al.’s (2009) study noted they did not have opportunities to collaborate on a regular basis, which made it hard for them to include an autistic student who used AAC.

###### Facilities and Materials

Some teachers described how they lacked the facilities and materials needed to include students (e.g., Downing et al., 2004). Across studies, GETs often described how including students was complicated by the ways their physical environment and curricula were inaccessible (e.g., Kent-Walsh & Light, 2003). For example, a first-grade teacher expressed concern that her curriculum “did not present options for adaptations” (Kent-Walsh & Light, 2003, p. 114). PE teachers described lacking necessary equipment, such as larger scooters, portable microphones, and ramps (Ko & Boswell, 2013). One PE teacher said, “I need something for students in wheelchairs to be able to use to grab their balloon when it goes to the floor” (Ko & Boswell, 2013, p. 235). Without basic supplies, such as reachers, accessible classrooms, books, and universally designed curricula, these teachers had to figure out other alternatives to adjust instruction, which took time (Kent-Walsh & Light, 2003; Ko & Boswell, 2013).

###### Informational Resources

Some educators described and appreciated systematic supports for learning about how to effectively serve their students. In Fisher et al.’s (2000) case study of a school that sustained inclusive reform during a time of substantial disruption, teachers spoke about the importance of extensive opportunities for helping them sustain inclusive practices. Their PD opportunities included biweekly after school in-service, district workshops twice/semester, and a summer program. As a result of what teachers had learned through PD, teachers felt able to continue the inclusive services, despite reductions in overall funding.

In contrast, many educators described lacking basic information about students and how to serve them effectively. In an inclusive charter school, a GET reflected on how they were just expected to know how to co-teach: “The expectation was we were going to be great at co-teaching, and we didn’t have the training to be great at co-teaching . . . . Collaboration was definitely hard sometimes” (Downing et al., 2004, p. 20). Other GETs described lacking basic information about students:

I’ve never been told that she has cerebral palsy, but she does. Though she has an educational assistant with her in the room, I know nothing–zero. If anything were to cause the educational assistant not to be there, I wouldn’t know the first thing about even pushing her wheelchair, much less getting her to understand or speak. (Kent-Walsh & Light, 2003, p. 112)

Another GET described being completely dependent on a paraeducator, who knew how to use a student’s AAC device (Kent-Walsh & Light, 2003). Few studies reported paraeducators’ experiences, but in Downing et al.’s (2004) study of an inclusive charter school, paraeducators also described lacking professional learning opportunities. One paraeducator reflected,

The only challenge I mainly had was what the kids needed. . .Who needed what, what you could and couldn’t do. . .I didn’t know that you can’t move this person’s pencil, or that they prefer to do certain things . . . . That kind of background knowledge would have been helpful. (Downing et al., 2004, p. 19)

## Discussion

We reviewed findings from 28 predominantly qualitative studies that explored educators’ perspectives in inclusive elementary schools. We found inclusion involved new responsibilities for principals and GETs; for example, principals took on responsibilities for setting an inclusion vision, building teachers’ capacity, reorganizing service delivery, and managing various conflicts (e.g., Keyes et al., 1999), while GETs assumed responsibilities for learning about each student, their disability, accommodating their needs in the classroom, and collaborating with SETs and service providers. Principals struggled with district policies, resistance, staffing, or funding mechanisms, while GETs needed PD, time for collaboration, and resources to address students’ needs. Of note, we identified no studies focused on educators’ working conditions when serving students with LD in inclusive settings—a glaring omission in the research.

### Critique and Recommendations for Future Research

Although we identified common themes across studies, the collective body of research is limited in many ways, reducing our capacity to draw conclusions about educators’ working conditions as they sought to include students with disabilities. Future reviews might include doctoral dissertations and reports, which could add additional insights into the educators’ working conditions. In this section, we describe the limitations within the collective body of research we reviewed, using them to suggest an agenda for future research.

#### Focus on Students With LD

Given that students with LD comprise the largest portion of students with disabilities in the United States, and that 75% of students with LD are included in general education classes (U.S. Department of Education, 2024), it is surprising that we found no research on the working conditions educators experience when teaching students with LD in inclusive settings. Some of our findings likely apply to educators’ efforts to serve students with LD; for example, we found that some of the working conditions that GETs experienced (e.g., insufficient PD, lack of time to plan and collaborate) likely affect their efforts to effectively teach students with LDs. Yet, additional research is needed to understand the specific working conditions necessary to effectively teach students with LD in inclusive settings. For example, researchers might examine how educators and administrators prioritize resources and supports for students with LD when students with more significant needs are in the same classrooms and how schools distribute resources for students with disabilities when schools confront budget cuts.

#### Focus on and Disaggregate Data for Different Educator Roles

Extant studies focused primarily on principals and GETs, though some included SETs and paraeducators (e.g., Hoppey et al., 2018). Studies of principals were stronger, providing in-depth insights into principals’ experiences of working conditions and how those conditions contributed to their decision-making (e.g., DeMatthews, 2015a). In some studies, principals described SETs’ activities, but typically did not include their perspectives (e.g., Keyes et al., 1999); in other studies, their perspectives were aggregated across participants (e.g., McLeskey et al., 2014). We also know little about the role of related services personnel and how they provide services in inclusive schools.

#### Investigate Supports for Productive and Effective Collaboration

The most consistent theme was the critical role of collaboration for educators’ work in inclusive schools. Principals relied on productive collaboration with district personnel and with school staff (Causton-Theoharis et al., 2011); teachers spoke about the importance of collaboration with one another (e.g., Lohrmann & Bambara, 2006); paraeducators described the need to collaborate with teachers (Causton-Theoharis et al., 2011); and researchers described how breakdowns in collaboration fractured service delivery for students (Kangas, 2017).

Across many of the studies, educators spoke about their difficulty finding time for collaboration. Given the lack of SETs’ perspectives, we know little about how SETs and GETs collaborated, thus future research needs to address how their responsibilities and resources were shared and how their interactions shaped their inclusive work. In a noteworthy exception, Kangas (2017) attended to the different roles SETs, EL teachers, and GETs played in supporting EL students with disabilities, yielding insights into how the construction of their roles left gaps in students’ service delivery. More studies are needed that carefully attend to the roles various educators play, and how working conditions shape their capacity to enact and coordinate their roles in ways that foster comprehensive, effective educational experiences for students.

Given that inclusion requires coordination of effort among educators across the school, future research on collaboration, as a critical responsibility and a resource for educators, is warranted. What organizational structures and scheduling strategies might they use to ensure educators have time for this critical responsibility? What norms and routines (e.g., using progress monitoring data) are productive for fostering stronger collaboration? What are the effects of different collaborative structures on the quality of services students’ experience? How can educators be prepared to engage in effective collaboration?

#### Make Linkages Among Working Conditions, Services, and Student Outcomes

A few studies referenced schools’ use of effective systems and practices (e.g., RtI, small group instruction, data-based decision-making; Causton-Theoharis et al., 2011; Hoppey et al., 2018; McLeskey et al., 2014). However, most studies described the working conditions educators appreciated or wanted, without making clear how these working conditions shaped the quality or effectiveness of the actual services they were providing, nor how those conditions might have shaped students’ experiences and outcomes. Thus, future research is needed that explicitly focuses on working conditions, and that allows researchers to make inferences about linkages among (a) working conditions, (b) educators’ provision of services to students, and (c) students’ experiences and learning.

Many of these studies included descriptions of supporting students with disabilities in inclusive settings through accommodations, modifications, and assistive technologies (e.g., Finke et al., 2009). What led some teachers to question whether inclusion was appropriate was a challenging behavior (e.g., Causton-Theoharis et al., 2011; DeMatthews et al., 2015b). However, we found no descriptions of the use of systematic behavioral supports in any of the studies we reviewed. Lohrmann and Bambara (2006) stated that GETs were not aware of systematic behavioral supports, thus they were not prepared to provide them. Educators in inclusive schools need to ensure students have meaningful access to the general education curriculum, while also meeting their ethical and legal responsibility to provide specialized instruction relevant to students’ individual IEP goals, yet specialized instruction was also seldom addressed as a responsibility, perhaps due to the limited focus on SETs.

#### Staffing Models and Division of Labor

Some of the research studies briefly described how schools organized staff for inclusion. For example, in Causton-Theoharis et al.’s (2011) study, each SET was assigned to two grade-level teams with two paraeducators; this type of organizational configuration reduced collaborative demands on teachers as they worked with a small group. Administrators are charged with making crucial decisions about staffing special education, including determining how many staff to hire, the qualifications of those staff, and how to systematically organize and support them. Yet, extant research provides very few insights into effective staffing models for inclusion. How many personnel, with what expertise, are needed to ensure all students’ needs are met? How should responsibilities for serving students be divided among these personnel—what divisions of labor are more, or less productive for maximizing each staff members’ expertise, without overloading them? These questions are core to leaders’ decision-making in establishing effective structures for inclusion, yet to date, they have been left almost totally unexamined.

#### Consider Contexts and Educators’ Beliefs About Inclusion

Further research is needed to understand broader contextual factors that shape the working conditions educators experience as they implement inclusion. Relevant contextual factors include district and state funding structures, district policies for supporting special education, partnerships with community groups, community engagement, and local instantiations of broader systems of oppression (e.g., racism). Understanding contextual conditions that give rise to more, or less supportive working conditions is needed for creating policies and practices that support educators to enact inclusion in all schools. For example, a principal’s strong values for inclusion influenced the responsibilities they assumed to create a more inclusive school, and their frustration with lack of resources to do so (e.g., insufficient staffing) (DeMatthews, 2015b). In contrast, no studies of teachers considered how their identities, values, or beliefs might have shaped their experiences of or responses to working conditions. For example, in Able et al.’s (2015) study, a teachers’ assumption that a students’ neurodivergent behaviors were responsible for the bullying they experienced did not consider how ableist social norms might be to blame, nor of their own responsibility to address the bullying and social isolation students experienced.

### Implications for Policy and Practice

First, districts aiming to be inclusive should carefully consider how they revise policies, staffing models, and resource allocations to ensure they are supporting all personnel (including school leaders, teachers, and paraeducators) in fulfilling their new responsibilities. Although PD is crucial (Fisher et al., 2000), teachers needed more time for collaboration and sufficient resources to meet the needs of students with disabilities in general education settings. Principal and educator support is also needed to encourage the use of specialized instruction and the use of data-based decision-making to provide each student’s team with information about how they are progressing and to inform necessary adjustments in instruction. Thus, policymakers and leaders need to provide schools with resources commensurate with the substantial structural changes they are asking schools to make.

Second, because inclusion requires coordination of effort across the school, studies often highlighted how principals are crucial for ensuring all educators are working together toward a shared goal. Yet many principals feel unprepared for this responsibility (Roberts & Guerra, 2017). Leadership development should consider guidance from the Professional Standards for Educational Leaders (National Policy Board for Educational Administration, 2015), and the supporting guidance document titled, *Promoting Principal Leadership for the Success for Students with Disabilities* (Council of Chief State School Officers and Collaboration for Effective Educator Development, Accountability, and Reform Center, 2017).

Third, many GETs felt unprepared for their roles in inclusive classes (e.g., Able et al., 2015; Lohrmann & Bambara, 2006). Thus, for some GETs, they were only beginning to learn on the job about how to address the needs of students with disabilities, sometimes with minimal support (e.g., Ko & Boswell, 2013). Given that 67% of students with disabilities are enrolled 80% or more of their time in general education (U.S. Department of Education, 2024), greater alignment is needed in between GET and SET preparation, with the goal of preparing all teachers to address the diverse needs of students (Blanton et al., 2018). Districts and schools also need to provide PD, with more targeted supports to GETs who enroll a new student with a disability (e.g., consultation with a specialist, regular collaborative support from SETs and related services personnel). Such efforts should be designed to help GETs understand students’ strengths, and their responsibility to create learning environments that respond to all students’ strengths and support needs.

## Conclusion

Inclusion is a complex, multifaceted process that takes place in organizations shaped by local, state, and national histories of inclusion and exclusion. Findings from this exploratory synthesis provide insights into the working conditions educators experienced in inclusive schools. We found inclusion changed educators’ responsibilities, yet many did not have the resources needed to implement it well, leaving them feeling stretched thin, and some struggled to meet students’ needs. Collaboration was an especially important responsibility, yet teachers described barriers to this crucial work. Our findings indicate a need for systematic and rigorous investigation of educators’ working conditions necessary to effectively meet their responsibilities to students with disabilities, including a specific focus on students with LD.

## Appendix

### Search Terms

Full Boolean search phrase used: (lead* OR principal* OR “school lead*” OR admin* OR direct* OR Dean* OR special educat* OR “general educator” OR “teach*” OR “classroom teach*”OR para* OR aide OR “teach* assistant” OR “speech therap*” OR “language therap*” OR “occupational therap*” OR therapist* OR psychol* OR counsel* OR “social work*”) AND (disab* OR impair* OR “hearing loss” OR deaf* OR dyslex*) AND (child* OR student*) AND (inclus* OR “inclus* class*” or “inclus* school”) AND (elementary OR primary OR intermediate OR kinder* OR “first grade*” OR “second grade*” OR “third grade*” OR “fourth grade*” OR “fifth grade*” OR “sixth grade*” OR “grade school”).
